# Effects of scapulothoracic exercises on chest mobility, respiratory muscle strength, and pulmonary function in male COPD patients with forward shoulder posture: A randomized controlled trial

**DOI:** 10.12688/f1000research.126832.2

**Published:** 2024-08-20

**Authors:** Kanogwun Thongchote, Usa Chinwaro, Sarawut Lapmanee

**Affiliations:** 1 Department of Physical Therapy, Faculty of Physical Therapy, Srinakharinwirot University, Nakorn Nayok, 26120, Thailand; 2 Department of Basic Medical Sciences, Faculty of Medicine, Siam University, Bangkok, 10160, Thailand

**Keywords:** COPD; scapulothoracic exercise; Respiratory muscles; Shoulders; Thoracic wall

## Abstract

Background

The postural abnormality, forward shoulder posture (FSP), is the most common cause of respiratory impairment in older individuals with chronic obstructive pulmonary disease (COPD). A recent study found that performing pectoral stretching and scapular strengthening exercises for eight weeks could reduce FSP in healthy participants. We aimed to determine the effects of pectoral stretching and scapular stabilizer strengthening exercises on FSP, chest wall mobility, respiratory muscle strength, and pulmonary function in male patients with COPD.

Methods

This study was randomized clinical trial. Forty male COPD patients with FSP aged 60–90 years were included and randomly allocated to control (n=20) and exercise (n=20) groups. Following completion of the scapulothoracic exercises (three days/week, for eight weeks), respiratory functions were assessed by measuring the magnitude of FSP, chest mobility, respiratory muscle strength, and pulmonary functions.

Results

FSP and thoracic kyphosis angle significantly decreased compared to controls (p<0.001, p<0.001). Middle and lower chest mobility markedly increased (p<0.001, p<0.001) and the pectoralis minor index significantly improved (p<0.001). The strength of the lower trapezius and serratus anterior muscles significantly increased at week eight of the exercise training (p<0.003, p<0.001). There was a marked increase in maximum inspiratory pressure and maximum expiratory pressure (p<0.001, p<0.001).

Conclusions

The eight-week combined pectoral muscles self-stretching and serratus anterior and lower trapezius strengthening exercises could be an effective treatment and/or prevention strategy for FSP reduction, leading to improved respiratory function in male COPD patients.

## Introduction

Chronic obstructive pulmonary disease (COPD) is one of the most important chronic inflammatory lung diseases that leads to increased mortality and morbidity worldwide. Globally, the prevalence of COPD is expected to increase, leading to an estimated 5.4 million deaths in 2060. ^1^ The proportion of COPD cases was higher in males (11.9%) than females (8.4%). ^2^ In Thailand, the prevalence of COPD was 2.1% in 1999 and increased to 7.0% in 2010. ^3^ Moreover, this disease incidence increases and outcomes deteriorate with age. ^4^ In COPD patients, hyperinflation of the lungs induces a passive increase in chest wall rigidity and respiratory muscle weakness, and also promotes postural misalignment i.e., forward shoulder posture (FSP) or rounded shoulders. ^5^^–^^7^

FSP is characterized by the resting shoulder position being located forward from the ideal postural alignment, which is linked to scapular protraction, anterior tilt, and downward rotation, as well as being positioned anteriorly. ^8^^,^^9^ This abnormal shoulder posture results in subacromial impingement and shoulder pain. Tightness of the pectoral muscles and weakness of the scapular muscles, especially the lower trapezius and serratus anterior, subsequently lead to a forward alignment of the shoulder posture. ^10^^,^^11^ A reduction in pulmonary function is related to an increase in FSP. ^12^ Therefore, poor posture resulting from FSP in COPD patients not only leads to musculoskeletal problems, but also causes deteriorating effects on the pulmonary system.

Many studies have suggested that exercises can improve muscle balance, and this is considered the treatment for FSP. ^13^^–^^15^ Stretching and strengthening exercises, especially for the serratus anterior and lower trapezius muscles, have been used to actively counteract the strength and movement loss associated with FSP. ^16^ Therefore, diminishing the muscle imbalance in FSP is an effective treatment for musculoskeletal dysfunction and for improving pulmonary function.

Improvements in rounded shoulders, respiratory muscle strength, and scapulothoracic muscle flexibility in COPD patients are beneficial for symptom management but do not directly correlate with changes in lung hyperinflation, lung function, dyspnea, or quality of life. This is due to the irreversible lung damage, fixed airway obstruction, and dynamic hyperinflation characteristic of COPD, which are not significantly altered by muscle strengthening alone. While these exercises enhance symptom management and functional capacity, they do not directly resolve the primary pathological changes of the disease, although they contribute to overall better management and slower progression of disability.

The primary gap in the study is the lack of research on the effects of pectoral stretching and scapular strengthening exercises on FSP and related respiratory impairments in older COPD patients. While these exercises have shown benefits in healthy individuals, their impact on COPD patients of varying severities remains underexplored. Our preliminary study suggested that an eight-week regimen of these exercises can alleviate FSP and improve chest mobility and respiratory muscle strength in individuals without COPD. Our preliminary study showed that an eight-week regimen of self-stretching the pectoral muscles and strengthening exercises for the scapular muscles can attenuate FSP, improve chest mobility, and increase respiratory muscle strength in participants without COPD. To our knowledge, this integrated exercise program was extended to COPD patients with FSP; however, the effects of these exercises have not been studied with older COPD patients. Therefore, this study aimed: ( *i*) to investigate the effects of an exercise training program comprising pectoral self-stretching and scapular strengthening exercises on FSP, and ( *ii*) to determine whether such an exercise rehabilitation program could also improve chest mobility, respiratory muscle strength, pulmonary function, and quality of life in geriatric patients with COPD.

## Methods

### Study design and subjects

A randomized controlled trial and single-blind study of all participants were performed. According to the Global Initiative for Chronic Obstructive Pulmonary Disease (GOLD) guidelines, stage 1–4 COPD patients were diagnosed by a respiratory medical doctor and trained spirometer personnel recruited from Nakhon Nayok Hospital and Banna Hospital in Nakhon Nayok province, Thailand. The study utilized an exercise protocol adjusted for each patient’s tolerance based on ACSM guideline, with the Rating of Perceived Exertion (RPE) limited to a maximum of 4/10. The exercise regimen included: weeks 1-2: 8-20 repetitions/set, 1 set/day; weeks 3-4: 10-22 repetitions/set, 2 sets/day; weeks 5-6: 12-24 repetitions/set, 3 sets/day; and weeks 7-8: 14-26 repetitions/set, 4 sets/day. Despite musculoskeletal pain and shoulder joint stiffness being listed as exclusion criteria, patients with such conditions were included in the study. Among COPD patients, the prevalence was higher in males than in females. ^2^ Therefore, forty male COPD patients with FSP aged 60–90 years were included, and their general appearance, personal history, and family history were characterized. A shoulder posture was forward if the distance from the anterior tip of the acromion process to the wall was longer than 2.54 cm or 1 inch. ^14^^,^^17^ Patients with acute exacerbation, neurological, or cardiovascular problems were excluded from the study.

The sample size was calculated from a study by Gaude *et al*. (2014) by using the G-power program (version 3.1.9.2, RRID:SCR_013726). ^18^ ANOVA with repeated measures was employed to determine interactions within the group (i.e., duration of intervention: baseline, week four and week eight) and interactions between groups (i.e., control vs exercise), which were used to calculate a statistical power of 0.8, α error probability of 0.05, and effect size of 0.38 was derived from a randomized trial over 12 weeks by Ghanbari A et al. (2008) using maximal inspiratory pressure (PImax) as a parameter for sample size calculations. ^12^ The sample size from the calculation was 16 participants per group. However, assuming that 20% would drop out equally, four additional participants were added per group. Therefore, two groups with 20 participants in each were conducted in the present study.

### Procedures

A flowchart of the process used in this study is provided in Figure Supplementary 1. Initially, 52 COPD (stage 1–4) patients were assessed for eligibility. Twelve volunteers were excluded from the study as they either declined to participate (n=1) or did not meet the inclusion criteria (n=11). The remaining 40 participants were randomly allocated into two groups: a control and an exercise group, with 20 participants in each. Two participants in the control group were excluded during the eight weeks as they exacerbated their COPD condition. In the exercise group, four participants were excluded in the fourth week due to COPD exacerbation (n=1) or discontinuation of their exercise intervention (n=3). However, the analysis of intention to treat with the last observation carried forward (LOCF) method was still conducted for them in this study. Theoretically, LOCF replaces missing outcomes with the last observed outcome. The intra-rater reliability of the tester was examined for all parameters. Each testing procedure was performed by the same investigator. The intra-class correlation coefficient (ICC _3,1_) was shown to lie between 0.960–0.997, which is considered a demonstration of an excellent level of reliability (p<0.01).

### Measurements of outcomes

Assessment of the primary outcomes included measurement of the magnitude of FSP, the amplitude of chest mobility, respiratory muscle strength, and pulmonary functions. These parameters are the gold standard measurements and tools for assessment of respiratory muscle strength, and pulmonary functions according to American Thoracic Society (ATS) recommendation. In addition, the assessment of the secondary outcomes included measurement of these items: pectoralis minor length, degree of thoracic kyphosis, serratus anterior and lower trapezius muscle strength, symptoms and quality of life, respectively. Assessment of outcomes was conducted at three time points: baseline at the start of the study, and again after four weeks and eight weeks of the training program.

### Primary outcome parameters

**Forward shoulder posture**

The magnitude of FSP was assessed using the vernier height gauge (Mitutoyo 506-207, Japan) in the sitting position. Measurement of the distance from the wall to the anterior aspect of the participant’s acromion process indicated the magnitude of FSP in centimeters. ^19^

**Chest wall mobility**

The amplitude of chest mobility was shown through the amplitude of the circumference of the thoracic wall during full expiration and inspiration. Three levels of chest wall circumference, upper, middle, and lower chest, were determined with apparent landmarks on the subjects’ skin. ^20^ A measuring tape was used to measure the level of chest expansion in centimeters.

**Strength of respiratory muscles**

Inspiratory and expiratory muscle strength was determined by the measurement of maximum voluntary inspiratory and expiratory pressures (MIP and MEP) in centimeters of water pressure (cmH _2_O) using a respiratory pressure meter (Micro RPM, Micro Medical Ltd., Rochester, UK). The evaluation followed the protocol of the American Thoracic Society/European Respiratory Society (ATS/ESR). ^8^^,^^21^ The measurement was performed for three to five maximal maneuvers within the range of 5–10% reproducibility and acceptability. ^22^

**Pulmonary functions**

According to the ATS/ESR guideline, spirometry was performed for a pulmonary function test in the sitting position. Volunteers were instructed to deeply inhale and forcefully exhale through a spirometer (Viasys Micro Lab 3500, UK) for a minimum of three maneuvers, but only eight repetitions. The acceptable repeatability of each maneuver followed the criteria of the ATS/ESR guideline. ^23^ The maximum values of forced vital capacity (FVC), forced expiratory volume within 1 sec (FEV1), and FEV1/FVC were recorded in percent predicted.

### Secondary outcome parameters

**Pectoralis minor length**

Left and right pectoralis minor lengths were measured using the vernier caliper (530-101 series, Mitutoyo, Japan) and the pectoralis minor index (PMI) was calculated. In a relaxed sitting position, markings were made on the inferior angle of the coracoid process and the fourth costosternal junction. On each side, the distance between these markers was measured in millimeters. ^24^

**Degree of thoracic kyphosis**

Postural thoracic kyphosis was evaluated using a flexible ruler. In the standing position, the spinous process of the seventh cervical vertebra and the twelfth thoracic vertebra were marked on the volunteer’s skin. At that point, the flexible ruler curved along the thoracic spines, and the thoracic angle was computed using the flexicurve method. ^9^

**Strength of scapulothoracic muscles**

Serratus anterior and lower trapezius muscle strength tests were performed, as described by previous studies. ^5^^,^^13^ The resistance force from the maximum isometric contraction was measured by the hand-held dynamometer (Baseline electronic push/pull dynamometer, Model 12-0342, Ufam Decoration Co., LTD) and was normalized by the body weight (in kilograms).

**Symptom of breathlessness**

The breathlessness score was assessed using a rate from the modified British Medical Research Council (mMRC). This method evaluates the characteristics the breathlessness which are related to health status and mortality risk. ^25^ An mMRC score<2 is identified as small breathlessness and an mMRC score≥2 is a large breathlessness.

**Quality of life**

Quality of life was assessed using the COPD assessment test (CAT). All participants were evaluated by CAT for quality of life. It is well accepted to have a good correlation with COPD health status measured by St. George’s Respiratory Questionnaire (SGRQ). The validity of the Thai CAT questionnaire was moderately correlated (r=0.652) with the quality of life questionnaire (SGRQ). The Thai CAT questionnaire has acceptable reliability and validity. It can be expected to serve as a short and simple tool for assessing the health status of Thai COPD patients. ^26^ The CAT has eight items including dyspnea, cough, sputum production, wheeze, systemic symptoms of fatigue, sleep disturbance, limitation in daily activities, social life, emotional health, and feeling control. CAT scores range from 0–40. A CAT score<10 is identified as having a low impact on health status, 11–20 is identified as a moderate impact on health status, 21–30 is identified as a high impact on health status, and >30 is identified as a very high impact to health status. ^27^

### Scapulothoracic exercise interventions

Each participant received routine conventional medical treatment, including physical therapy with breathing control using the pursed-lip technique. For the control group, the conventional physical therapy treatment consisted of pursed-lip breathing exercises, education on relaxation techniques, and training in coughing and huffing. These interventions were provided to both groups. For the exercise group, the participants’ vital signs were measured before exercise and then during exercise, as shown in Figure Supplementary 2. The exercise protocol in this study was supervised and applied to all participants by the same investigator. The participants followed an exercise training program for reducing FSP that was modified from previous studies. ^28^^–^^30^ The exercise plan comprised pectoral muscles self-stretching at 90° and 120° of shoulder abduction with external rotation and serratus anterior and lower trapezius strengthening exercises. The exercises were done three days a week for eight weeks.

For the stretching exercise, Participants engaged in two pectoral muscle stretching exercises. For the pectoralis major (clavicular part) and pectoralis minor, they adopted a standing position behind a white line with shoulders abducted to 90° and elbows flexed to 90°. They performed the stretch by rotating the trunk to the opposite side, holding the position for 60 seconds at the end range of motion, while breathing through pursed lips. This exercise was carried out 5 times per set, 1 set per day, 3 days per week, for 8 weeks. For the pectoralis major (sternal costal part), participants stood behind a yellow line with shoulders abducted to 120º and elbows flexed, stretching in a similar manner but performing 10 repetitions per set. This was done with five repetitions/set, one set/day, 60 seconds for holding, and a 30-second break between each stretch. ^31^

For the lower trapezius strengthening, a scapular posterior tilting exercise was performed in a prone sitting position, as followed in the previous studies. ^28^^,^^30^^,^^32^ Push-ups on stable table support were modified from a study by Kisner and Colby in 2013 for strengthening the serratus anterior. ^28^

### Statistical analysis

Intention-to-treat analysis with the LOCF method was performed in this study. The results were expressed as mean±SE and were analyzed by the IBM SPSS program version 25 (SCR_002865). An unpaired t-test was used to test the characteristics of the participants at baseline. Two-way repeated measures ANOVA was used for comparison of the main effects of all parameters between baseline, week four, and week eight of the exercise training. The level of significance of the statistical tests was set at p<0.05.

## Results

### Demographic data of COPD patients at baseline and patient characteristics

The demographic data of COPD participants in the control and exercise group are shown in Table 1. There was no significant difference in any of the characteristics of the subjects, which were age, body mass index (BMI), weight, height, number of participants in each stage of COPD, CAT for quality of life, and mMRC for breathlessness score (p>0.05). The baseline characteristics of all participants in the control and exercise groups are shown in Table 1. There were no significant differences in the baseline data between the control and the exercise groups, which reflects the success of the randomization in creating the intervention and control groups with similar baseline characteristics. The interaction between time and intervention was significantly shown in the magnitude of FSP, MIP, and MEP (p<0.05).

**Table 1.** Demographic data and baseline characteristics of participants (mean±SE).

Characteristics		Control (n=20)	Exercise (n=20)	p value
**Age** (years)		71.10±1.02	70.80±1.00	0.83
**Body mass index**; BMI (kg/m ^2^)		21.84±0.60	22.22±0.66	0.68
**Weight** (kg)		59.39±1.80	58.38±1.80	0.69
**Height** (cm)		164.90±1.31	162.25±1.69	0.22
**Duration of disease** (years)		9±1.71	8±0.79	0.59
**Duration of disease** (min–max; years)		1–31	1–15	0.59
**Smoking** (pack/year)		28.78±4.05	25.75±3.79	0.58
**COPD stage** ^**a**^ (n)				
I		1	3	
II		13	12	0.71
III		4	4	
IV		2	1	
**CAT** ^a^				0.70
- Low impact (< 10 point)		17	15	
- Moderate impact (10–20 points)		3	5	
- High impact (21–30 points)		0	0	
- Very high impact (> 30 points)		0	0	
**mMRC** ^a^				
- Low impact (<2 points)		14	11	
- High impact (≥2 points)		6	9	0.51
**Forward shoulder posture**; FSP (cm)	Dominant	7.6±0.24	7.3±0.35	0.52
	Non-dominant	7.0±0.27	6.7±0.27	0.47
**Pectoralis minor index**; PMI	Dominant	9.61±0.13	9.62±0.14	0.99
	Non-dominant	9.93±0.13	9.78±0.12	0.40
**Muscle strength test**				
Force of lower trapezius (N/kgbw)	Dominant	1.64±0.99	1.74±0.10	0.48
Force of serratus anterior (N/kgbw)		3.66±0.2	3.45±0.2	0.49
Force of lower trapezius (N/kgbw)	Non-dominant	1.46±0.11	1.58±0.08	0.40
Force of serratus anterior (N/kgbw)		3.36±0.18	3.16±0.17	0.41
**Thoracic kyphosis** (degree)		39.93±2.16	40.57±1.97	0.83
**Chest expansion**				
- Upper part (cm)		3.2±0.15	3.4±0.15	0.45
- Middle part (cm)		3.6±0.17	3.9±0.17	0.29
- Lower part (cm)		4.7±0.27	4.7±0.28	0.90
**Respiratory muscle strength**				
- MIP (cmH _2_O)		70.40±3.72	65.80±2.24	0.30
- MEP (cmH _2_O)		95.10±5.23	90.30±4.35	0.49
**Pulmonary function test**				
- FVC (%predicted)		74.05±4.29	81.30±3.27	0.19
- FEV1 (%predicted)		56.85±3.72	59.05±4.32	0.70
- FEV1/FVC (%predicted)		73.15±3.78	68.80±4.38	0.46

COPD stage was identified according to the Global Initiative for Chronic Obstructive Lung Disease (GOLD) guideline.

cm, centimeters; N/kgbw, newtons per kilogram of body weight; cmH _2_O, centimeters of water; FVC, forced vital capacity; FEV1, forced expiratory volume in one second; MIP, maximum inspiratory pressure; MEP, maximum expiratory pressure.

^a^ Fisher’s exact test.

Effects of eight-week scapulothoracic exercises on FSP, PMI, lower trapezius and serratus anterior muscle strength, and thoracic kyphosis.

**Figure 1.**
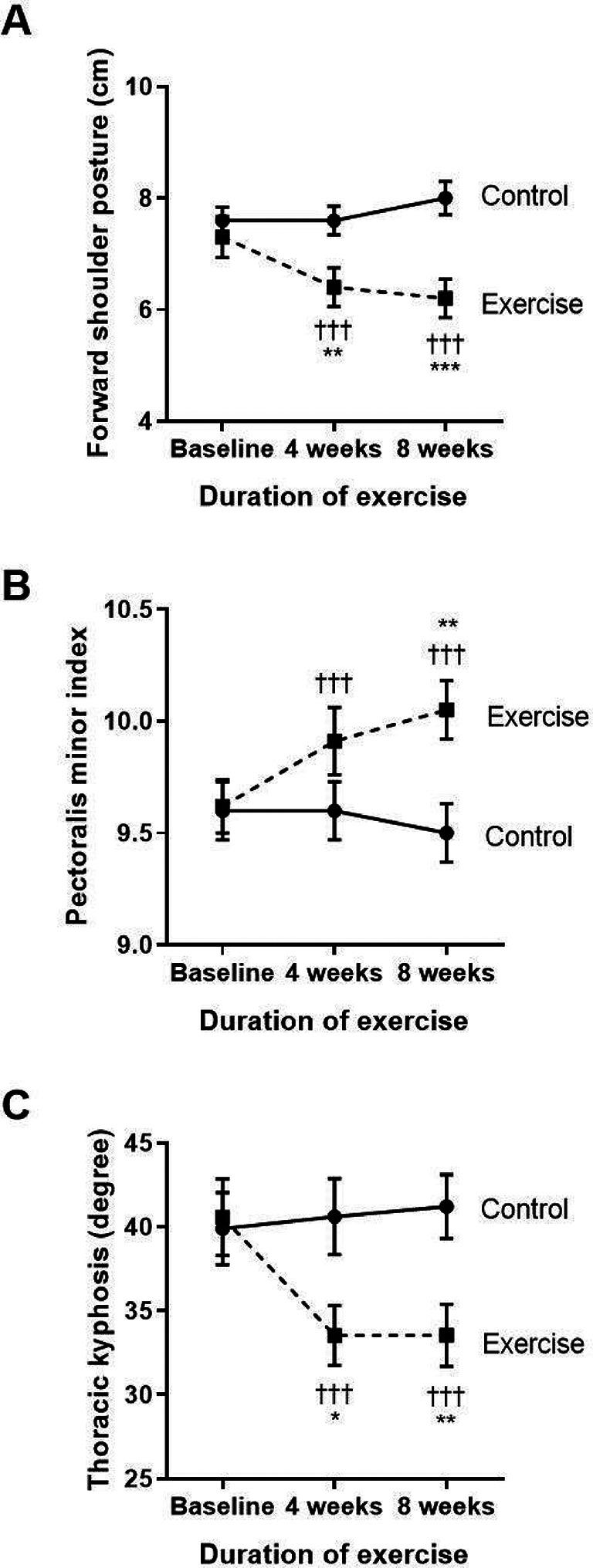
Comparison of the degree of forward shoulder posture (A), pectoralis minor index (B), and thoracic kyphosis (C) between the control group and exercise group at baseline, and at four and eight weeks of the study period. †††p<0.001 compared to baseline, *p<0.05 compared to control, **p<0.01 compared to control, ***p<0.001 compared to control. cm=centimeters.

**Figure 2.**
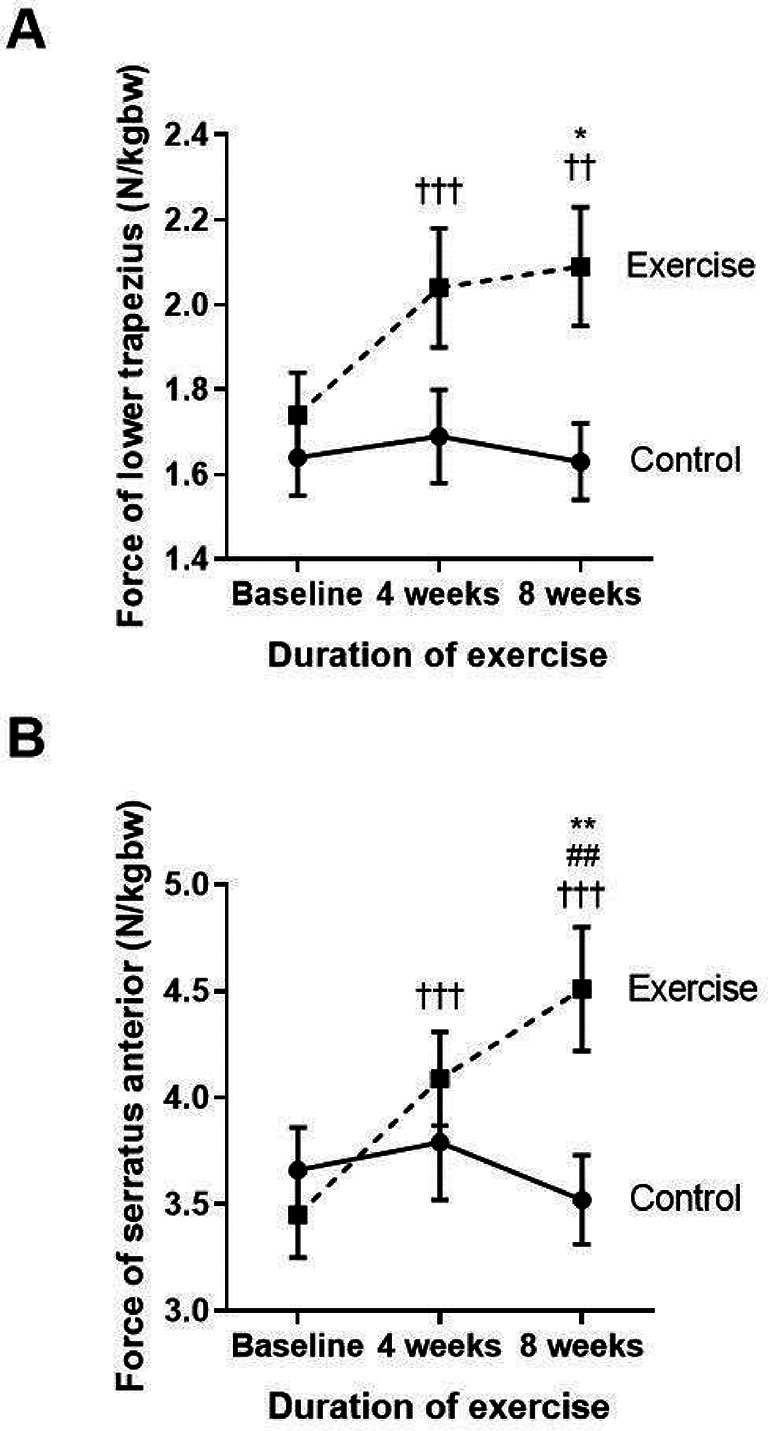
Comparison of the muscle force in the lower trapezius (A), and serratus anterior (B) between the control group and exercise group at baseline, and at four and eight weeks of the study period. ††p<0.01 compared to baseline, †††p<0.001 compared to baseline, ##p<0.01 compared between four weeks and eight weeks, *p<0.05 compared to control, **p<0.01 compared to control. N/kgbw=newtons per kilogram of body weight.

The key results of the side measurements, including both within-group and between-group findings of the structural changes for FSP, PMI, thoracic kyphosis, and strength of lower trapezius and serratus anterior muscles are shown in Figures 1 and 2. Regarding the changes in FSP within each group ( Figure 1A), the control group showed a significantly increased FSP at week eight when compared to baseline, whereas the exercise group had a reduction in FSP at both weeks four and eight when compared to baseline (p<0.001, p<0.001, respectively) and compared to the control (p<0.01, p<0.001, respectively). These results show that FSP gradually increased with time in COPD patients without exercise, whereas performing the eight-week exercise training could ease FSP in COPD patients from week four to week eight of performing the exercise training.

As shown in Figure 1B, there were no changes in PMI within the control group. However, the exercise group showed significantly improved PMI when compared to their baseline at week four (p<0.001) and week eight (p<0.001) of the training. There was also an incremental increase of PMI at week eight (p=0.008) when compared to the control.

Regarding thoracic kyphosis ( Figure 1C), the exercise group displayed a significantly reduced degree of thoracic kyphosis at week four (p<0.001) and week eight (p<0.001) when compared to baseline. These results demonstrate that the exercise program improved the alignment of the thoracic spine by improvement of the degree of thoracic kyphosis from week four to week eight of the exercise training.

The two major scapular stabilizer muscles, namely the lower trapezius and serratus anterior, were evaluated ( Figure 2A and B). When compared to the baseline, the exercise group demonstrated a significantly increased amount of force generated by the lower trapezius and serratus anterior muscles at week four (p<0.001; p<0.001) and at week eight (p=0.003; p<0.001), whereas the force was not changed in the control group. Additionally, there was a marked increase in lower trapezius and serratus anterior muscle force observed at week eight of the training when compared with the control group (p=0.01; p=0.09).

These results show that reducing FSP by performing eight weeks of self-stretching the pectoral muscles and scapular muscle strengthening exercises, including the lower trapezius and serratus anterior, can improve the pectoralis minor muscle length. This is represented by PMI and the scapular muscle strength, especially in the lower trapezius and serratus anterior muscles.

### Effects of eight-week scapulothoracic exercises on chest wall mobility

**Figure 3.**
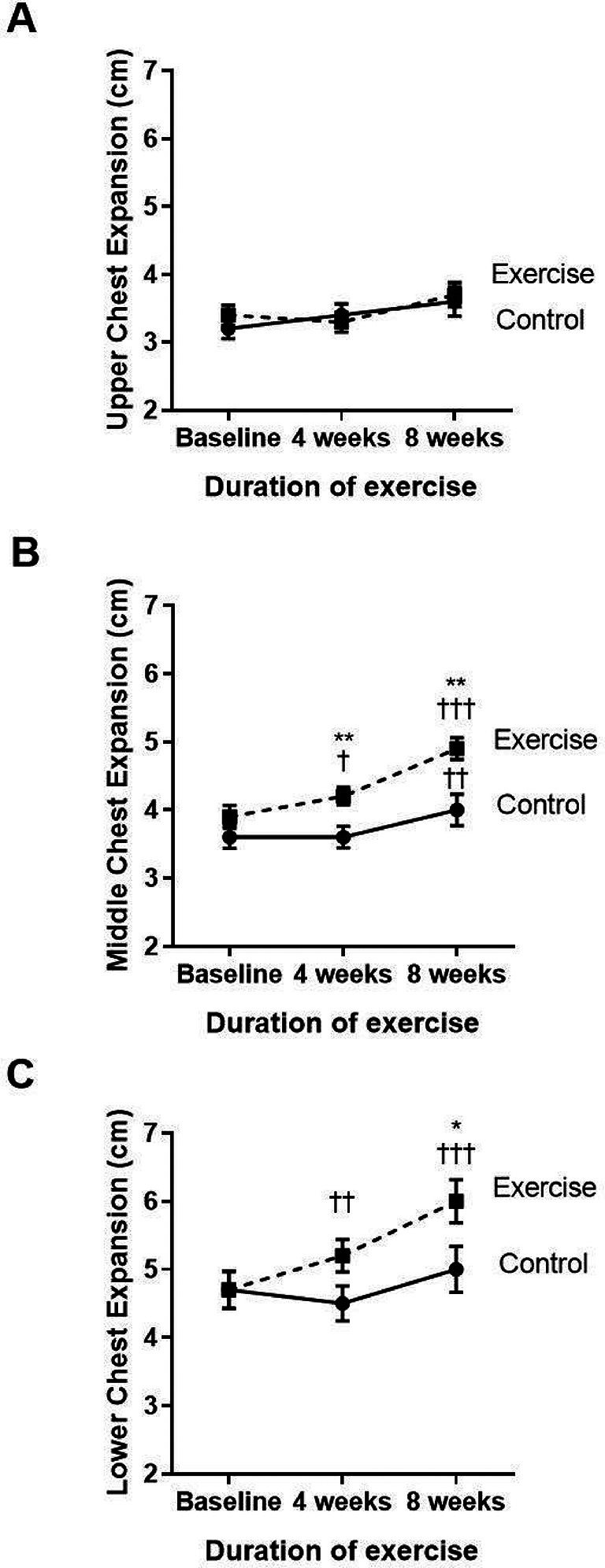
Comparison of upper (A), middle (B), and lower chest expansion (C) between the control group and exercise group at baseline, and at four and eight weeks of the study period. †p<0.05 compared to baseline, ††p<0.01 compared to baseline, †††p<0.001 compared to baseline, *p<0.05 compared to control, **p<0.01 compared to control. cm=centimeters.

The three parts of the chest wall, including the upper, middle, and lower parts, were evaluated regarding chest mobility or expansion ( Figure 3A– C). Compared to baseline, the exercise group showed significantly increased middle (p<0.001) and lower (p<0.001) chest expansion at week four and week eight of the training course. When compared to the control (p=0.039), the exercise group showed an incremental increase in middle chest expansion in weeks four and eight, whereas the lower chest showed improvement only at week eight. However, the upper chest expansion did not show any statistically significant change during the four to eight weeks of training exercises.

### Effects of eight-week scapulothoracic exercises on respiratory muscle strength

**Figure 4.**
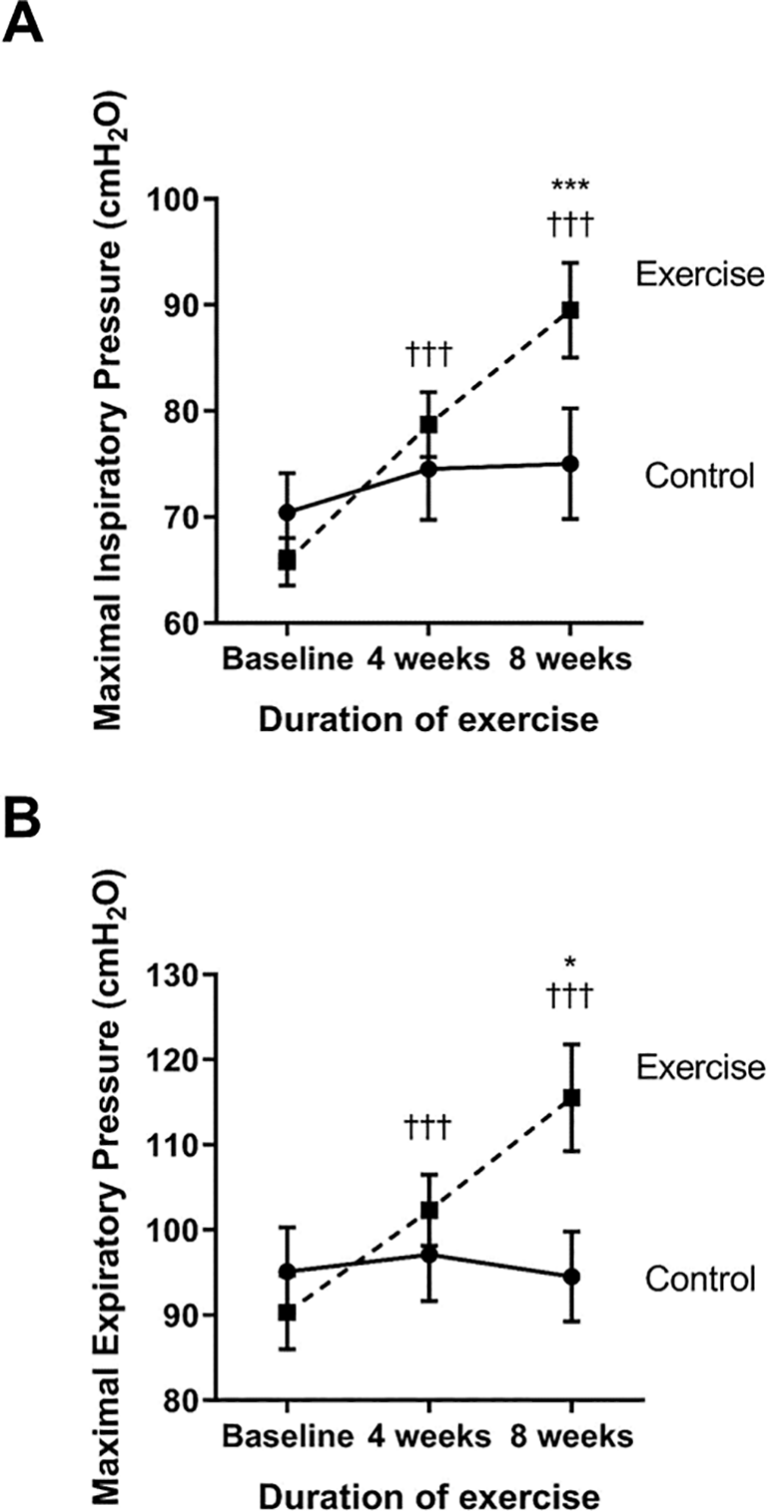
Comparison of maximal inspiratory pressure; MIP (A) and maximal expiratory pressure; MEP (B) between the control group and exercise group at baseline, and at four and eight weeks of the study period. †††p<0.001 compared to baseline; *p<0.05 compared to control; ***p<0.001 compared to control. cmH _2_O=centimeters of water.

The strength of the inspiratory and expiratory muscles was measured as the maximal inspiratory (MIP) and expiratory pressure (MEP), respectively (see Figure 4A and B). Compared to baseline, the exercise group showed a significantly increase in MIP and MEP at week four (p<0.001 and p<0.001, respectively) and week eight (p<0.001 and p<0.001, respectively). When compared to the control, the MIP and MEP of the exercise group were significantly increased at week eight (p<0.001 and p<0.001, respectively) through the training exercises.

### Effects of eight-week scapulothoracic exercises on pulmonary functions

Regarding the pulmonary function test, the exercise group did not display a significant difference in the pulmonary function parameters: the %FVC predicted, %FEV _1_ predicted, and %FEV _1_/FVC when compared with baseline and with the control group, as shown in Table Supplementary 1.

### Effects of eight-week scapulothoracic exercises for reducing FSP on mMRC, and CAT

Considering the mMRC and CAT score, breathlessness (mMRC) and quality of life of the participants with COPD (CAT) were not changed in the exercise group when compared within the group and with the control, as illustrated in Table Supplementary 2. These results imply that an eight-week exercise training program could not change the patients’ quality of life. However, the training program did not induce the severity of breathlessness in the older participants with COPD.

## Discussion

This study evaluated the effects of an eight-week exercise regimen for reducing FSP on chest expansion, respiratory muscle strength, and pulmonary function in COPD patients. The results showed that performing an exercise training program that included stretching pectoral muscles and strengthening scapular muscles three days a week for eight weeks improved chest expansion and respiratory muscle strength.

In the exercise group, there was a significant reduction in the degree of FSP with an increase in PMI. Similarly, soft tissue mobilization and stretching of the pectoralis muscles could reduce FSP, which led to alleviation of scapular anterior tilting in the sagittal plane and scapular internal rotation in the transverse plane. ^20^ In addition, the viscoelastic effect of muscle stretching increases the range of motion, which is related to a reduction in the resistance to stretch and muscle stiffness, and an increase in muscle compliance. ^11^ Reducing tension in the stretched pectoral muscles allows them to lengthen and also decreases FSP.

To correct muscle imbalance, the strengthening exercises for the agonist’s muscles are important to counteract muscle weakness related to FSP. The lower trapezius and serratus anterior are the primary agonist muscles to control posterior tilting and upward rotation of the scapular, which are necessary for the subacromial space expansion in FSP. ^33^ Therefore, activation of the lower trapezius and serratus anterior muscles is important for correcting muscle imbalance and re-establishing the normal plane of the scapula in COPD patients with FSP.

Our results showed a significant increase in the magnitude of lower trapezius and serratus anterior muscle force in the exercise group. Therefore, eight-week scapular strengthening exercises focusing on the lower trapezius and serratus anterior muscles after stretching the pectoral muscles could be applied as a potential method to increase the length of the pectoralis minor muscle and the strength of the scapular muscles. An increase in scapular stabilizing muscle strength could adjust the proprioception of shoulder joint and restore the scapular position to the normal scapula-humeral alignment, thus reducing FSP in COPD patients. ^34^ These findings are consistent with the eight-week exercise program, which is comprised of stretching the anterior shoulder muscles (i.e., pectoralis, levator scapulae, and sternocleidomastoid) and strengthening of the posterior shoulder muscles (i.e., middle trapezius, lower trapezius, and serratus anterior) that could decrease FSP in swimmers. ^35^

This study showed that the correction of the upper quadrant muscles in COPD patients improved, not only shoulder posture, but also the posture of the thoracic spine. These results are consistent with the findings of Kim *et al*.(2018) who reported that rehabilitation with resistive and stretching exercises could re-balance muscle force and restore muscle elasticity, thus reducing FSP and thoracic kyphosis angle. ^36^

Some recognize that poor posture with FSP limits respiratory function. Alteration of upper body alignment changes respiratory muscle length, especially the diaphragm and intercostal muscles, affecting its force-generating capacity. ^37^ The tightness of the pectoralis muscle resulting from FSP limits the mobility and compliance ability of the chest wall to create maximal lung capacity during inspiration. ^23^ COPD patients with FSP have a reduced ability to raise and expand the thorax, which limits their lung capacity. ^32^ Regarding FSP reduction, pectoralis muscle stretching causes the pectoralis muscle fibers to lengthen, as shown by the increased PMI, and the compliance of the anterior chest wall is improved.

Our findings showed an improvement in chest wall mobility when compared with the control. The increment in chest mobility was shown in the middle section, which is the area of the pectoralis minor muscle. Reduction of FSP provides the approximation of the ribs to the pelvis, decreases intra-abdominal pressure, and allows the diaphragm to descend caudally. ^14^ Presumably, the diaphragm can work more effectively to expand the lower chest and create maximum contraction force during respiration, as indicated in the increased lower chest mobility and respiratory muscle strength found in this study.

Considering the pulmonary function, the exercises for reducing FSP led to no significant improvement of the pulmonary functions, including FEV _1_, FVC, and FEV _1_/FVC. This indicates that correcting FSP does not alleviate the respiratory abnormalities in COPD patients. Our findings are consistent with a study by Wang (2015), in which no noticeable changes were found in the respiratory functions (i.e., FEV _1_, FVC, and FEV _1_/FVC) of COPD patients after posture was improved by stretching and mobilization of the thoracic cage. ^38^ The explanation for the non-improvement of the pulmonary function in the COPD participants was reported as being due to the advanced age, advanced thoracic kyphosis, and the longer duration of disease in the participants. In addition, smoking history is associated with the severity of airway inflammation in COPD patients. These factors result in structural deterioration, making it difficult to restore.

The prevention of aggravated symptoms was also found in this study as it was illustrated that there were no changes in the breathlessness score and quality of life in the exercise-trained COPD participants when compared to their control. Our findings are consistent with a study by McKeough (2016), in which no significant decrease in dyspnea in a patient with stable COPD was reported after upper training. ^39^ However, in a meta-analysis study, upper limb training was given in a shorter period of three to eight weeks and there was a significant decrease in dyspnea, suggesting that a short duration of upper limb training can reduce dyspnea in stable COPD patients. ^40^ Quality of life was assessed by CAT score and there were no changes in the exercise-trained COPD participants when compared to their control. Consequently, this study only shows that upper limb training improves or corrects shoulder posture in stable COPD but does not improve the quality of life in a patient with COPD. ^39^

Overall, improvements in rounded shoulders, respiratory muscle strength, and scapulothoracic flexibility in COPD patients may not directly correlate with changes in lung function, dyspnea, and quality of life due to several factors. COPD is characterized by irreversible lung damage that exercises alone cannot rectify. Pulmonary function tests primarily assess mechanical aspects of lung function and may not reflect changes in muscle strength or posture. ^41^ Dyspnea and quality of life are influenced by a range of factors, including psychological and comorbid conditions, and improvements in muscle function might not significantly affect overall symptoms. ^42^ Additionally, established compensatory mechanisms and limitations of measurement tools might obscure subtle exercise-induced changes. ^43^ Thus, while targeted exercises can enhance specific aspects of physical function, significant improvements in lung function, dyspnea, and quality of life often require a comprehensive management approach for lung diseases. ^44^^,^^45^

The limitations of this study included the reduction in FSP found in this study resulting from the combination of a static self-stretching and strengthening exercise program. Firstly, differential analysis between pectoral stretching and scapular muscle strengthening exercises should be performed in future studies. Secondly, the findings of this study cannot be generalised to other situations or conditions, such as female patients or males with other pulmonary diseases, as the present study was only comprised of 60 to 90-year-old male COPD patients. Thirdly, the detraining effects after the eight-week exercise program could be observed in order to observe respiratory muscular adaptations. Finally, only a single-blind study was performed in the present research because of the limited number of investigators. To minimize bias, the blinding of all therapists and assessors should be considered in any future research.

## Conclusions

Based on these findings, the eight-week regimen of pectoral stretching and scapular strengthening exercises could improve pectoral muscle tightness and scapular muscle strength i.e., lower trapezius and serratus anterior muscles, which can lead to significantly reduced FSP in male COPD patients. There was increased chest mobility and respiratory muscle strength following this exercise intervention. Thus, the results might provide perspectives for the improvement of the quality of life in male COPD patients.

## Ethical approval

This study was approved by the Institutional Review Board of the Nakhon Nayok Hospital (REC 10/2560) and the Nakhon Nayok Public Health Office (NPHO 2018-004) by the World Medical Association’s Declaration of Helsinki.

## Informed consent

Data collection and the need for informed consent were approved by the Thai Clinical Trial Registry (registration number TCTR20180525001). Written informed consent was obtained from each of the participants.

## Author contributions

Conceptualization, KT, SL; Data curation, KT, UC, SL; Formal Analysis, KT; Funding acquisition, KT, SL; Investigation, KT, UC, SL; Methodology, KT, UT; Visualization, KT, SL; Project administration, KT; Supervision, SL; Writing–original draft, KT, SL; Writing–review & editing, UC, SL, KT.

## Submission statement

All authors have read and agree with manuscript content. The manuscript has not been published and is not under consideration for publication elsewhere.

## Data Availability

Figshare: [Additional methods], https://doi.org/10.6084/m9.figshare.21312333. The project contains the following underlying data:
- Methods Data repository name: [Supplementary data]. https://doi.org/10.6084/m9.figshare.21312378. This project contains the following extended data:
-
**Supplementary Table 1.** Group comparison of pulmonary functions at baseline (week zero), week four, and week eight of the study period. -
**Supplementary Table 2.** COPD assessment test (CAT) and modified Medical Research Council (mMRC) dyspnea scale; comparison at baseline, and at four and eight weeks of the study period. -
**Supplementary Figure 1.** Flow diagram for the study. -
**Supplementary Figure 2.** Exercise intervention comprised of pectoral stretching and scapular stabilizer exercise. Pectoralis major (clavicular part) and pectoralis minor stretching exercise: (A and B) starting position, (C and D) stretch at the end range of motion. Scapular stabilizer exercises: (E) serratus anterior exercise push-up on table, scapular posterior tilt (SPT) exercise: (F) starting position (G) SPT in long arm. (H) Progression of strengthening exercise protocol was modified from ACSM’s prescription. -
**Questionnaire: appendix form**. Data are available under the terms of the Creative Commons Zero “No rights reserved” data waiver (CC0 1.0 Public domain dedication).
