# Supplementary material for: Effects of scapulothoracic exercises on chest mobility, respiratory muscle strength, and pulmonary function in male COPD patients with forward shoulder posture: A randomized controlled trial
Source: F1000Res. 2024 Aug 20;11:1284. Originally published 2022 Nov 10. [Version 2] doi: 10.12688/f1000research.126832.2 (PMC11393525; doi:10.12688/f1000research.126832.2)
Supplement: Peer review history

# Reviewer report: Reviewer response for version 2

**DOI:** 10.5256/f1000research.170003.r324569

**Published:** 2024-09-30

**Authors:** Gopal Nambi

**Affiliations:** 1 Prince Sattam Bin Abdul Aziz University, Alkharj, Saudi Arabia

The article is not suitable to publish in its current state. 

 Abstract:

1. The title and the objective of the study is contrary.
2. Mention the eligibility criteria of the study participants.
3. Mention the intervention provided to the participants in short.
4. The outcome measures used in the study are not sufficient.
5. Mention the statistical tests used for the study.
6. Present the reports with 95%CI (Confidence interval) with upper and lower limits for all outcome variables.
7. The conclusion of the study is not clear; it should be drawn on the basis of study reports.

Manuscript:

1. Please elaborate the introduction part as it is not scientifically written. 
2. Provide a reference for scapula thoracic exercise for COPD patients.
3. Mention the application procedure, merits, and demerits of scapula thoracic exercise for COPD patients.
4. The need for the study is not mentioned clearly in recent references.
5. Mention the gaps monitored by the researcher in the previous studies.
6. Include the clinical significance of the study for the researchers, clinicians and patients.
7. Mention the study design, study duration and study setting.
8. Include the ethical approval and clinical trial registration number.
9. Follow the CONSORT guidelines to present the study.
10. Mention the character of the study participants.
11. Mention the randomization and allocation procedure in clear.
12. Mention the blinding procedure in detail.
13. Include the diagnostic criteria of the disease and its ICD classification.
14. Mention who has diagnosed the participants and their qualifications and experience.
15. Mention the outcome variables with their reliability and validity.
16. Include the figures for the intervention program, for study replication.
17. Mention the intervention parameters of study interventions.
18. Include the sample size calculation with a suitable reference.
19. The samples included in the study are not sufficient to generalize the results.
20. The statistical tests included are not apt for this study.
21. Mention how the co-variables are controlled and how the homogeneity is measured.
22. Mention the treatment compliance rate, dropout rate and adverse effects.
23. Present the reports with 95%CI with upper and lower limits for all outcome variables.
24. Include the MCID score and effect size of all the variables.
25. Summarize the discussion part.
26. Include the mechanism behind the changes in the outcome variables with these intervention modalities in these patients.
27. The conclusion of the study is not clear; it should be drawn based on study reports.
28. Include the real-time limitations faced by the researcher.
29. Include the future recommendations of the study. 

Is the work clearly and accurately presented and does it cite the current literature?

Partly

If applicable, is the statistical analysis and its interpretation appropriate?

No

Are all the source data underlying the results available to ensure full reproducibility?

No

Is the study design appropriate and is the work technically sound?

No

Are the conclusions drawn adequately supported by the results?

No

Are sufficient details of methods and analysis provided to allow replication by others?

No

Reviewer Expertise:

NA

I confirm that I have read this submission and believe that I have an appropriate level of expertise to state that I do not consider it to be of an acceptable scientific standard, for reasons outlined above.

# Reviewer report: Reviewer response for version 2

**DOI:** 10.5256/f1000research.170003.r315480

**Published:** 2024-09-12

**Authors:** Pattanasin Areeudomwong

**Affiliations:** 1 Department of Physical Therapy, School of Integrative Medicine, Mae Fah Luang University, Chiang Rai, Thailand

The authors improved the manuscript significantly. I appreciate their update, but I suggest adding more data about the results of the repeated-measures ANOVA, such as interaction and main effects, to highlight noteworthy findings.

Is the work clearly and accurately presented and does it cite the current literature?

Partly

If applicable, is the statistical analysis and its interpretation appropriate?

No

Are all the source data underlying the results available to ensure full reproducibility?

Yes

Is the study design appropriate and is the work technically sound?

Yes

Are the conclusions drawn adequately supported by the results?

Yes

Are sufficient details of methods and analysis provided to allow replication by others?

Partly

Reviewer Expertise:

Exercise therapy, Manual therapy, Musculoskeletal Physiotherapy

I confirm that I have read this submission and believe that I have an appropriate level of expertise to confirm that it is of an acceptable scientific standard.

# Reviewer report: Reviewer response for version 1

**DOI:** 10.5256/f1000research.139281.r243958

**Published:** 2024-02-29

**Authors:** Sonia Pawaria

**Affiliations:** 1 Faculty of Physiotherapy, SGT University, Gurugram, Haryana, India

The randomized controlled trial examined the effects of scapulothoracic exercises on male COPD patients with forward shoulder posture. Through a comprehensive assessment, including measures of chest mobility, respiratory muscle strength, and pulmonary function, the study aimed to elucidate the potential benefits of targeted interventions. Participants underwent a structured exercise regimen focusing on scapulothoracic region mobility and strength. The results revealed significant improvements in chest mobility, indicating enhanced thoracic expansion. Additionally, there were notable enhancements in respiratory muscle strength, suggesting improved respiratory efficiency. Pulmonary function assessments demonstrated favourable changes, with notable increases in vital capacity and forced expiratory volume. These findings underscore the potential of scapulothoracic exercises as an adjunct therapy for COPD patients with forward shoulder posture, offering a promising avenue for optimizing respiratory health and functional outcomes. However, further research is warranted to explore the long-term effects and optimal dosage of such interventions. I would like to acknowledge the commendable effort undertaken by the researchers in investigating posture correction strategies in COPD. Currently, pulmonary rehabilitation predominantly emphasises breathing retraining, making this exploration particularly significant.

Is the work clearly and accurately presented and does it cite the current literature?

Yes

If applicable, is the statistical analysis and its interpretation appropriate?

Yes

Are all the source data underlying the results available to ensure full reproducibility?

Yes

Is the study design appropriate and is the work technically sound?

Yes

Are the conclusions drawn adequately supported by the results?

Yes

Are sufficient details of methods and analysis provided to allow replication by others?

Yes

Reviewer Expertise:

Pulmonary Rehabilitation, Women Health, Renal Rehabilitation

I confirm that I have read this submission and believe that I have an appropriate level of expertise to confirm that it is of an acceptable scientific standard.

# Reviewer report: Reviewer response for version 1

**DOI:** 10.5256/f1000research.139281.r166096

**Published:** 2023-03-27

**Authors:** Pattanasin Areeudomwong

**Affiliations:** 1 Department of Physical Therapy, School of Integrative Medicine, Mae Fah Luang University, Chiang Rai, Thailand

This study looked at the effects of scapulothoracic muscle stretching and strengthening exercises on COPD outcome outcomes. Although the major sources of chest wall problems, such as barrel chest shape, which commonly occurs in COPD patients, are not addressed directly in this work, it is interesting to apply simple interventions to address the consequences of COPD in terms of scapulothoracic muscle imbalance. My suggestions for improving this manuscript are as follows:

**Title:**

- "A randomized control trial" should be "a randomized controlled trial" in title of the study. Please double-check the manuscript for spelling, typos, and grammatical errors.

**Abstract:**

- When compared to the control group, it is unclear which intervention improves outcome measures. Please explain such intervention in the results section.

**Introduction:**

- In COPD patients, lung hyperinflation reduces chest wall mobility and causes poor posture, such as rounded shoulders and scapulothoracic muscle imbalance. Because the main problems of chest wall rigidity, respiratory muscle weakness, and lung hyperinflation are not solved by the interventions in this study, pectoral muscle stretching and lower trapezius and serratus anterior muscle strengthening exercises may temporarily improve such COPD problems. As a result, as I mentioned in the introduction section, the authors should provide possible mechanisms of used interventions for improving outcomes.
- Please explain the study's gap and why it is important to examine current interventions in older COPD patients at various stages of COPD.

**Methods**

- For the study, researchers enrolled COPD patients in stages 1-4. Do the various stages of COPD affect patients' exercise performance and tiredness?
- Are COPD patients with musculoskeletal problems, such as pain and shoulder joint stiffness, excluded from the study?
- What parameter is used to estimate an effect size of 0.38?
- How many times does the assessor measure each outcome?
- Please double-check whether the paper cited at number 9 in the list proposes a method of measuring thoracic kyphosis.
- On Page 5, I do not believe that breathing control with pursed lips is merely a routine conventional medical and physiotherapy treatment for COPD.
- Please rewrite pectoral muscle stretching. Is each arm fixed for the purpose of stretching?
- Why don't the researchers use two-way repeated measures ANOVA instead of two-way ANOVA for the three outcome measurement periods?

**Results:**

- Page 7: What are " *the key results of the side measurements...*"?
- Because the figures show both within- and between-group findings, "within-group comparison" should be indicated in all figure captions.

**Discussion:**

- Please provide additional reasons why improvements in rounded shoulders, respiratory muscle strength, and scapulothoracic muscle flexibility and strength in COPD patients do not correlate with unchangeable lung function, dyspnea, and quality of life.

Is the work clearly and accurately presented and does it cite the current literature?

Partly

If applicable, is the statistical analysis and its interpretation appropriate?

No

Are all the source data underlying the results available to ensure full reproducibility?

Yes

Is the study design appropriate and is the work technically sound?

Yes

Are the conclusions drawn adequately supported by the results?

Yes

Are sufficient details of methods and analysis provided to allow replication by others?

Partly

Reviewer Expertise:

Exercise therapy, Manual therapy, Musculoskeletal Physiotherapy

I confirm that I have read this submission and believe that I have an appropriate level of expertise to confirm that it is of an acceptable scientific standard, however I have significant reservations, as outlined above.

# Response

**Published:** 2024-08-03

**Authors:** Sarawut Lapmanee

**Affiliations:** Siam University, Thailand

**Title:**

 - "A randomized control trial" should be "a randomized controlled trial" in title of the study. Please double-check the manuscript for spelling, typos, and grammatical errors.

      Thank you for your suggestion. We have already used "a randomized controlled trial" in the title of the study. Additionally, we have double-checked the manuscript for spelling, typos, and grammatical errors.

**Abstract:**

 - When compared to the control group, it is unclear which intervention improves outcome measures. Please explain such intervention in the results section.

      We have made the correction as per your suggestion by adding "In the COPD group with combined pectoral muscles self-stretching and scapular strengthening exercise" at the beginning of the results section of the abstract.

**Introduction:**

 - In COPD patients, lung hyperinflation reduces chest wall mobility and causes poor posture, such as rounded shoulders and scapulothoracic muscle imbalance. Because the main problems of chest wall rigidity, respiratory muscle weakness, and lung hyperinflation are not solved by the interventions in this study, pectoral muscle stretching and lower trapezius and serratus anterior muscle strengthening exercises may temporarily improve such COPD problems. As a result, as I mentioned in the introduction section, the authors should provide possible mechanisms of used interventions for improving outcomes.

       Improvements in rounded shoulders, respiratory muscle strength, and scapulothoracic muscle flexibility and strength in COPD patients are important for managing the disease but do not necessarily correlate with changes in lung hyperinflation, lung function, dyspnea (shortness of breath), and quality of life. Several reasons and possible mechanisms of used interventions for improving outcomes due to nature of COPD.

      1. Irreversible Lung: Damage COPD involves chronic and irreversible damage to the lung tissues and airways. While muscle exercises can improve the mechanics of breathing, they cannot reverse the structural damage or inflammation within the lungs.

      2. Fixed Airway Obstruction: The primary issue in COPD is the persistent airflow limitation caused by airway narrowing and destruction of alveoli. This limitation cannot be significantly altered by improving muscle strength and flexibility.

      3. Hyperinflation: During physical exertion, COPD patients often experience dynamic hyperinflation, where trapped air in the lungs increases, further reducing the efficiency of breathing. This condition is not directly affected by improvements in muscle strength or posture.

      4. Secondary Gains: While improvements in muscle strength and chest wall mobility do not directly change barrel chest shape, lung hyperinflation, they can lead to better management of symptoms, enhanced exercise tolerance, and potentially slower progression of disability. These benefits, while significant, may not be immediately reflected in traditional measures of lung function or quality of life scales.

      In summary, while scapulothoracic muscle balance exercises offer significant benefits in terms of posture, muscle strength, and flexibility, they do not directly address the primary pathological changes in COPD that drive lung hyperinflation, lung function decline, dyspnea, and overall quality of life. However, they play a crucial role in the comprehensive management of the disease, contributing to better symptom management and functional ability.

 - Please explain the study's gap and why it is important to examine current interventions in older COPD patients at various stages of COPD.

      The primary gap identified in the study is the lack of research focusing on the effects of pectoral stretching and scapular stabilizer strengthening exercises on forward shoulder posture (FSP) and associated respiratory impairments specifically in older patients with chronic obstructive pulmonary disease (COPD). Previous studies have demonstrated the benefits of these exercises in healthy individuals, but there is limited evidence regarding their impact on COPD patients, particularly across different severities of the disease.

      Importance of examining interventions across all severity of COPD

      1. Variable impact of COPD: COPD affects individuals differently depending on the severity of the disease. Patients with mild COPD might have different rehabilitation needs compared to those with severe COPD. Understanding how these exercises benefit patients across the entire spectrum of disease severity is crucial for developing tailored treatment plans.

      2. Personalized rehabilitation programs: By examining the effects of these exercises in patients with varying COPD severities, clinicians can better personalize rehabilitation programs. This ensures that each patient receives the most appropriate and effective interventions for their specific condition.

      3. Comprehensive care: COPD is a progressive disease, and interventions effective at one stage might not be as effective at another. Investigating the benefits of pectoral stretching and scapular strengthening exercises across all severity levels can provide insights into the most effective timing and intensity of these interventions.

      4. Maximizing clinical outcomes: Effective management of COPD requires strategies that can address the wide range of symptoms and complications associated with the disease. By understanding how these exercises impact respiratory function, chest mobility, and muscle strength at different stages, healthcare providers can maximize clinical outcomes.

      5. Informed decision-making: Robust evidence on the efficacy of these exercises across all severities of COPD will aid clinicians in making informed decisions about incorporating these exercises into routine care. This can lead to more consistent and evidence-based practices in the management of COPD.

      6. Preventative and therapeutic benefits: Identifying the benefits of these exercises in both early and advanced stages of COPD can help in formulating preventative strategies as well as therapeutic interventions. Early intervention might slow disease progression, while continued exercise in later stages can help maintain function and reduce complications.

      Therefore, addressing the study gap by investigating the effects of pectoral stretching and scapular strengthening exercises in older COPD patients across all severities of the disease is crucial. It will help develop comprehensive, evidence-based rehabilitation strategies that improve respiratory function, reduce FSP for COPD patients at every stage of their condition.

**Methods:**

 - For the study, researchers enrolled COPD patients in stages 1-4. Do the various stages of COPD affect patients' exercise performance and tiredness?

      No, the various stages of COPD did not affect patients' exercise performance and tiredness in this study because we adjusted the exercise protocol, modified from ACSM 2014, for each patient based on their tolerance. We also controlled the Rating of Perceived Exertion (RPE) to not exceed 4/10.

 - Are COPD patients with musculoskeletal problems, such as pain and shoulder joint stiffness, excluded from the study?

      No, COPD patients with musculoskeletal problems were not excluded from this study, even though musculoskeletal pain and shoulder joint stiffness were listed in the exclusion criteria.

  - What parameter is used to estimate an effect size of 0.38?

      We used an effect size of 0.38 derived from a randomized trial over 12 weeks by Ghanbari A et al. (2008) from maximal inspiratory pressure (PImax) as a parameter for sample size calculations.

 - How many times does the assessor measure each outcome?

      Assessment of the outcomes was performed three times. Baseline measurements were performed at the start of the study and were tested after four weeks and after eight weeks of the training program.

 - Please double-check whether the paper cited at number 9 in the list proposes a method of measuring thoracic kyphosis.

      Thank you for your feedback. We would like to clarify that reference number 9 ((Borstad and Ludewig, 2005) correctly addresses the position of FSP and is not cited for measuring thoracic kyphosis.

 - On Page 5, I do not believe that breathing control with pursed lips is merely a routine conventional medical and physiotherapy treatment for COPD.

      Conventional physical therapy treatment for the control group consisted of pursed-lip breathing exercises, education on relaxation techniques, and training in coughing and huffing.

 - Please rewrite pectoral muscle stretching. Is each arm fixed for the purpose of stretching?

      Pectoral muscles self-stretching exercise

      1.1 Stretching pectoralis major muscle **(**clavicular part **) **and pectoralis minor muscle

      - The participants were positioned in a standing position behind white line, with 90º shoulder abduction and 90º elbow flexion at corner as shown in Figure 1 **(**A **)**

      - The participants actively stretch pectoral muscle by moving trunk rotation to the opposite side as shown in Figure 1 **(**B **)**

      - Sustain a stretching for 60 seconds the end range of motion without pain **. **Fully breathe in and out with pursed lip **. **Then, return to starting position **.**

      - Stretching exercise will be performed 5 times **/**set, 1 set **/**day, 3 days **/**week, for 8 weeks

      1.2 Stretching pectoralis major muscle (sternal costal part) 

      - The participant will be positioned in a standing position behind yellow line, with 120º shoulder abduction and elbow flexion at the corner as shown in Figure 2 (A)

      - The participants actively stretch pectoral muscle by moving trunk rotation to the opposite side as shown in Figure 2 (B).

      - Sustain a stretching for 60 seconds the end range of motion without pain. Fully breathe in and out with pursed lip. Then, return to staring position.

      - Stretching exercise will be performed 10 times/set, 1 set/day, 3 days/week, for 8 weeks

 - Why don't the researchers use two-way repeated measures ANOVA instead of two-way ANOVA for the three outcome measurement periods?

      We have revised our analysis to use two-way repeated measures ANOVA for the three outcome measurement periods, as recommended.

**Results:**

 - Page 7: What are "the key results of the side measurements..."?

      Because the figures show both within- and between-group findings, "within-group comparison" should be indicated in all figure captions.

**Discussion:**

 - Please provide additional reasons why improvements in rounded shoulders, respiratory muscle strength, and scapulothoracic muscle flexibility and strength in COPD patients do not correlate with unchangeable lung function, dyspnea, and quality of life.

      Improvements in rounded shoulders, respiratory muscle strength, and scapulothoracic muscle flexibility and strength in COPD patients may not correlate with unchangeable lung function, dyspnea, and quality of life for several reasons:

      1. Disease Pathophysiology: COPD is primarily characterized by irreversible airflow limitation due to chronic inflammation, alveolar destruction, and airway remodeling. While exercises can improve muscle strength and posture, they do not directly address the underlying structural damage to the lungs.

      2. Nature of Lung Function Tests: Pulmonary function tests, such as FEV1 (Forced Expiratory Volume in one second) and FVC (Forced Vital Capacity), primarily measure the mechanical aspects of lung function, which are less likely to be influenced by changes in muscle strength or posture. These tests reflect the extent of airway obstruction and lung damage rather than the efficiency of respiratory muscles.

      3. Dyspnea and Quality of Life Factors: Dyspnea (shortness of breath) and quality of life in COPD patients are influenced by a complex interplay of factors, including psychological aspects, overall physical conditioning, comorbidities, and the extent of lung damage. While improved muscle strength and posture can alleviate some symptoms, they may not be sufficient to significantly alter the overall perception of breathlessness and life quality.

      4. Compensatory Mechanisms: COPD patients often develop compensatory mechanisms to cope with their condition, such as altered breathing patterns and muscle use. Improvements in muscle strength and posture might not be enough to overcome these established patterns and significantly change lung function or symptom perception.

      5. Measurement Sensitivity: The tools used to measure lung function, dyspnea, and quality of life might not be sensitive enough to detect subtle changes resulting from exercise interventions. Improvements in muscle function and posture might contribute to better breathing mechanics and reduced symptoms, but these changes might not be captured by standard clinical assessments.

      6. Multifactorial Nature of COPD: COPD affects multiple systems in the body, including the cardiovascular and muscular systems. While targeted exercises can improve specific aspects of physical function, comprehensive management involving medication, nutrition, and other interventions is necessary to achieve significant improvements in overall lung function, dyspnea, and quality of life.

 Therefore, while targeted exercises can lead to significant improvements in posture, muscle strength, and flexibility, the multifactorial and chronic nature of COPD means that these changes may not directly translate to improvements in lung function, dyspnea, and quality of life, which are influenced by a broader range of factors.

 References

 1. Global Initiative for Chronic Obstructive Lung Disease (GOLD). (2023). *Global Strategy for the Diagnosis, Management, and Prevention of Chronic Obstructive Pulmonary Disease (2023 Report)*. Retrieved from GOLD Website.

 2. Dodd, J. E., Horne, J. M., & McKeon, C. (2020). “The effectiveness of exercise training in the management of chronic obstructive pulmonary disease: a systematic review.” *Respiratory Medicine*, 166, 105951. doi:10.1016/j.rmed.2020.105951.

 3. Janssens, W., et al. (2019). "The Impact of COPD on Quality of Life: The Role of Clinical and Psychological Factors." *Chest*, 155(5), 947-955. doi:10.1016/j.chest.2018.12.023.

 4. Dixon, A. E., & Holguin, F. (2020). "The importance of the musculoskeletal system in COPD: Why this matters." *Thorax*, 75(8), 649-651. doi:10.1136/thoraxjnl-2019-214911.

 5. O'Donnell, D. E., et al. (2019). "The effect of respiratory muscle training on dyspnea and quality of life in patients with COPD." *Chest*, 155(5), 1147-1156. doi:10.1016/j.chest.2019.01.014.

 6. Gosselink, R., et al. (2021). "Exercise training in patients with COPD: A review of the evidence." *International Journal of Chronic Obstructive Pulmonary Disease*, 16, 2231-2242. doi:10.2147/COPD.S307680.

 7. Bourbeau, J., & Julien, D. (2021). "Impact of COPD on patient’s quality of life." *European Respiratory Review*, 30(162), 210045. doi:10.1183/16000617.0045-2021.

 8. Brady, S. L., et al. (2019). "Effect of pulmonary rehabilitation on health-related quality of life in COPD: A systematic review and meta-analysis." *Respiratory Care*, 64(10), 1296-1305. doi:10.4187/respcare.06839.

 9. Koulouris, N. G., & Makris, D. (2018). "The role of respiratory muscle strength in chronic obstructive pulmonary disease." *Breathe*, 14(1), e24-e32. doi:10.1183/20734735.001418.

 10. Palange, P., et al. (2020). "Multidisciplinary approach to chronic obstructive pulmonary disease: Role of exercise training." *Respiration*, 99(7), 568-576. doi:10.1159/000504163.
